# Three-Dimensional Source Localization with Sparse Symmetric Cross Array

**DOI:** 10.3390/s22134949

**Published:** 2022-06-30

**Authors:** Haowei Wu, Yiqiao Shi, Jinglan Ou

**Affiliations:** 1 School of Microelectronics and Communication Engineering, Chongqing University, Chongqing 400044, China; wuhaowei@cqu.edu.cn (H.W.); shiyiqiao@cqu.edu.cn (Y.S.); 2 Chongqing Key Laboratory of Space Information Network and Intelligent Information Fusion, Chongqing 400044, China

**Keywords:** three-dimensional (3-D) source localization, reduced-dimension MUSIC (RD-MUSIC), sparse symmetric cross array, DOA estimation

## Abstract

Three-dimensional (3-D) localization information, including elevation angle, azimuth angle, and range, is important for locating a single source with spherical wave-fronts. Aiming to reduce the high computational complexity of the classical 3-D multiple signal classification (3D-MUSIC) localization method, a novel low-complexity reduced-dimension MUSIC (RD-MUSIC) algorithm based on the sparse symmetric cross array (SSCA) is proposed in this article. The RD-MUSIC converts the 3-D exhaustive search into three one-dimensional (1-D) searches, where two of them are obtained by a two-stage reduced-dimension method to find the angles, and the remaining one is utilized to obtain the range. In addition, a detailed complexity analysis is provided. Simulation results demonstrate that the performance of the proposed algorithm is extremely close to that of the existing rank-reduced MUSIC (RARE-MUSIC) and 3D-MUSIC algorithms, whereas the complexity of the proposed method is significantly lower than that of the others, which is a big advantage in practice.

## 1. Introduction

Recent years have witnessed a massive rapid development in near-field source localization with increasing applications in various scenarios, such as radars, sonar systems, and vehicle networks. The source localization is one of the key techniques for target detection in radars [1,2]. In underwater sonar systems, the location of submerged sources is usually regarded as parameter estimation in the near field due to the relatively large array aperture [3,4]. In the field of Internet of Vehicles, the direction of arrival (DOA) estimation of landmark signal also belongs to one type of the near-field source localization [5,6]. Therefore, the research of near-field signal source localization and parameter estimation algorithm has a comprehensive application perspective and practical value. Near-field sources are located in the Fresnel region of the array [7] and their wave-fronts are in the form of spheres, which are different from plane waves of far-field sources [8]. Thus, localization algorithms for near-field sources have to yield DOA information as well as range parameters.

Several algorithms have been proposed for estimating the locations of near-field sources, such as the multiple signal classification (MUSIC) algorithm [9,10], estimation of signal parameters via rotational invariance techniques (ESPRIT) algorithm [11,12], maximum likelihood estimation (MLE) algorithm [13], and propagator method (PM) [14]. Among these algorithms, the MUSIC algorithm takes full advantage of all the information contained in the received signal, i.e., the source signal and noise components, which yields highly accurate parameters and super-resolution DOA results. However, the MUSIC algorithm is computationally intensive due to its searching in the global spatial domain. In order to reduce the computational complexity of the classical MUSIC algorithm, a lot of work has been completed in existing studies. A two-dimensional (2-D) polynomial root finding method for the near-field source localization was proposed in [15], which integrates the search in the distance domain with polynomial rooting and replaces the search in the azimuth angle with polynomial rooting. A rank-reduced MUSIC (RARE-MUSIC) algorithm [16] and a reduced-dimension MUSIC optimization algorithm [17] based on the uniform linear array were presented, where both algorithms decompose the direction matrix into the form of multiplying two matrices to reduce the implementation cost by searching the matrix only containing the angle term instead of a two-dimensional spectrum search.

A three-dimensional (3-D) parameters estimation of the source can provide elevation, azimuth, and range metrics to obtain accurate spatial location information of the source, which is suitable for practical requirements. Most of the existing 3-D localization algorithms are based on uniform circular array (UCA) and cross array (CA) structures. The UCA structure has 360 degree azimuth coverage, nearly constant directional diagram, and additional elevation information. Based on the UCA structure, the path tracing method is used to extract location information of near-field sources from the 3-D MUSIC spectrum [18], and the computational burden is large due to the need for 3-D spectral peak searching, which cannot be applied in engineering. A computationally simple algorithm based on a UCA for simultaneous estimation of the 3-D locations of a single source was proposed in [19]. Although this method has low computational complexity, it is only suitable for UCA structures with an even number of sensors. The single-source 3-D localization algorithm using the UCA structure was presented in [20], where the Fourier transform is utilized to extend the phase distribution of the single source, and the minimum number of array elements of the UCA structure is required to set to be 5, according to the Fourier sampling theorem. For the CA structure, there is an advantage that many 2-D near-field source localization algorithms can be extended to the 3-D case, since their array manifolds satisfy the form of the Vandermonde Matrix. A 3-D source location algorithm based on a center-symmetric CA was developed in [21], where only second-order statistics are adopted, leading to insufficient estimation accuracy. In [22], a localization method utilizing high-order cumulants was investigated, which can distinguish mixed far- and near-field sources with high time consumption.

Aiming at the problems that the number of sensors used in the uniform array structure is large and the algorithm is computationally intensive in the 3-D source localization, a low-complexity reduced-dimension MUSIC (RD-MUSIC) algorithm is introduced with sparse symmetric cross array (SSCA) in this article. The proposed SSCA structure is composed of two vertically cross coprime symmetric linear arrays, each of which consists of two uniform linear arrays with elements deployed at mutually prime spacing on the identical horizontal line. This simple structure can not only avoid localization ambiguity but also improve spatial resolution. Compared with the uniform symmetric cross array (USCA), the SSCA has a larger array aperture when the number of array elements is identical, which can improve the estimation accuracy effectively. From another angle, under the same array apertures, the SSCA contains fewer array elements than the USCA, which can reduce the complexity of the receiver.

Moreover, the proposed RD-MUSIC algorithm is performed by following three steps. Firstly, the 3-D joint spectral search is divided into two 2-D estimation problems through the relationships between the direction matrices of the x-axis subarray and the y-axis subarray. Secondly, to obtain angle parameters, the 2-D estimation problems are simplified into two one-dimensional (1-D) spectral searches, by decomposing the direction matrices of the subarrays on the x- and y- axes. Finally, the range can be obtained by substituting the angles into the original 3-D spectral search. In summary, the RD-MUSIC algorithm transforms the extensive search of the traditional three-dimensional MUSIC (3D-MUSIC) algorithm into three 1-D searches, which greatly reduces the computational complexity. Simulation results verify the effectiveness of the proposed RD-MUSIC algorithm.

The remaining parts of the article are organized as follows. Section 2 presents the system model. In Section 3, the proposed RD-MUSIC algorithm with SSCA is described in detail. Section 4 analyzes and compares the computational complexity of various algorithms. In Section 5, simulation results are given for the RD-MUSIC algorithm compared with the Cramer-Rao bound (CRB) and other algorithms. The conclusions are provided in Section 6.

In this article, matrices and vectors are represented by capital letters and lower case letters in boldface, respectively. Furthermore, the notations used are summarized in Table 1.

## 2. System Model

**Figure 1:**
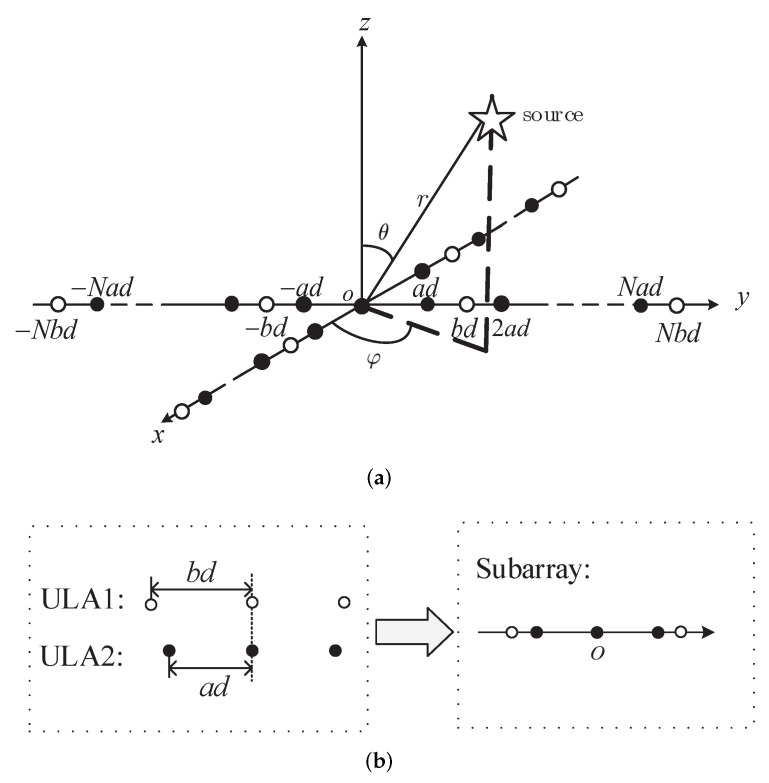
SSCA model. (**a**) SSCA with single source (**b**) Subarray of SSCA in an axis.

The SSCA model is shown in Figure 1, which is different from the classical ULA and USCA models. Instead of the uniform spacing in classical ULA or USCA, the coprime inter-element spacing is configured in SSCA to form the coprime linear array and further to establish the sparse symmetric cross array structure. Figure 1a shows the geometry of the SSCA, which contains two identical subarrays that are vertical to each other. Only one element of the two subarrays overlaps at the origin marked as “o”, which is also the reference point of the cross array.

The composition of a subarray is indicated in Figure 1b, where two uniform linear arrays, ULA1 and ULA2, are superimposed, which are denoted by “∘” and “•”, respectively. ULA1 and ULA2 are symmetric ULAs with array element spacing bd and ad, respectively, where *a* and *b* are coprime integers $\left(a<b\right)$, d=λ/4, and λ denotes the wavelength of the incident signal. Assuming a subarray contains *M* omnidirectional sensors, the positions of all sensors in an axis are in the set {−Nbd, $-Nad,\left(-N+1\right)bd,\left(-N+1\right)ad,\cdots ,0,\cdots ,Nad,Nbd\}$, where N=M−1M−144.

The SSCA with 2M−1 sensors is impinged by a single narrowband signal from the source. From the view of the array, the source is assumed to be located at a position referring to the origin of the array, denoted as three parameters, i.e., the azimuth angle φ∈(−π,π) counterclockwise from the *x*-axis, the elevation angle θ∈(0,π/2) measured down from the *z*-axis, and the distance *r* between the source and the origin. Therefore, the goal of source localization is to find the tuple of these parameters $\left\{\theta ,\varphi ,r\right\}$.

The signal received at the *m*-th sensor of *x*-axis and *y*-axis can be modeled as

(1) $$x_{m}\left(t\right)=e^{j\tau_{x,m}}s\left(t\right)+n_{x,m}\left(t\right)$$

(2) $$y_{m}\left(t\right)=e^{j\tau_{y,m}}s\left(t\right)+n_{y,m}\left(t\right)$$

respectively, where $s\left(t\right)$ denotes the source signal; $n_{x,m}\left(t\right)$ and $n_{y,m}\left(t\right)$ stand for the independent Gaussian white noise at the *m*-th sensor of *x*-axis and *y*-axis, respectively. The value range of the index *m* is $\left\{\left.-2N,-2N+1,\cdots ,0,\cdots ,2N\right\}\right.$.

The phase shifts in (1) and (2), corresponding to the signal propagation delay from the phase reference point to the *m*-th sensor on the *x*-axis and *y*-axis, are expressed as

(3) $$\tau_{x,m}=\frac{2\pi r}{\lambda }\left(\sqrt{1+{\left(\frac{l_{x,m}d}{r}\right)}^{2}-\frac{2l_{x,m}d\sin\theta \cos\varphi }{r}}-1\right)$$

(4) $$\tau_{y,m}=\frac{2\pi r}{\lambda }\left(\sqrt{1+{\left(\frac{l_{y,m}d}{r}\right)}^{2}-\frac{2l_{y,m}d\sin\theta \cos\varphi }{r}}-1\right)$$

respectively, where $l_{x,m}$ and $l_{y,m}$ denote the distances from sensors to the origin point.

In order to obtain the simplification of phase shifts, an intermediate function is built to denote the squared root term in (3), which is given by

(5) $$\begin{array}{c} g\left(\beta \right)=\sqrt{1+{\left(l_{x,m}\beta \right)}^{2}-2l_{x,m}\beta \sin\theta \cos\varphi } \end{array}$$

where β=ddrr. Then, by means of the Taylor series expansion, we can obtain the following expression

(6) $$\begin{array}{cc} g\left(\beta \right)= & g\left(0\right)+g^{\prime }\left(0\right)\beta +\frac{1}{2!}g^{\prime \prime }\left(0\right)\beta^{2}+\frac{1}{3!}g^{\prime \prime \prime }\left(0\right)\beta^{3}+\cdots \\ = & 1-\left(l_{x,m}\sin\theta \cos\varphi \right)\beta +\frac{1}{2}l_{x,m}^{2}\left(1-\sin^{2}\theta \cos^{2}\varphi \right)\beta^{2}+o\left[\beta^{3}\right] \end{array}$$

where $g^{\prime }\left(0\right)$, $g^{\prime \prime }\left(0\right)$ and $g^{\prime \prime \prime }\left(0\right)$ represent the value of the first, second, and third derivative of $g\left(\beta \right)$ at β=0, respectively. When β≪1, the higher order term $o[\beta^{3}]$ in the above equation can be ignored. Therefore, $\tau_{x,m}$ can be approximated as

(7) $$\begin{array}{c} \tau_{x,m}\approx -\frac{2\pi l_{x,m}d\sin\theta \cos\varphi }{\lambda }+\frac{\pi l_{x,m}^{2}d^{2}\left(1-\sin^{2}\theta \cos^{2}\varphi \right)}{\lambda r}=l_{x,m}\gamma_{x}+l_{x,m}^{2}\phi_{x} \end{array}$$

where $\gamma_{x}=-\left(2\pi d\sin\theta \cos\varphi \right)/\lambda$, and ϕx=πd21−sin2θcos2φπd21−sin2θcos2φλr(λr).

Similarly, $\tau_{y,m}$ can be approximated as

(8) $$\begin{array}{c} \tau_{y,m}\approx -\frac{2\pi l_{y,m}d\sin\theta \sin\varphi }{\lambda }+\frac{\pi l_{y,m}^{2}d^{2}\left(1-\sin^{2}\theta \sin^{2}\varphi \right)}{\lambda r}=l_{y,m}\gamma_{y}+l_{y,m}^{2}\phi_{y} \end{array}$$

where $\gamma_{y}=-\left(2\pi d\sin\theta \sin\varphi \right)/\lambda$, and $\phi_{y}=\pi d^{2}\left(1-\sin^{2}\theta \sin^{2}\varphi \right)/\left(\lambda r\right)$.

To this end, (1) and (2) can be rewritten to be matrix form, as

(9) $$x\left(t\right)=a_{x}s\left(t\right)+n_{x}\left(t\right)$$

(10) $$y\left(t\right)=a_{y}s\left(t\right)+n_{y}\left(t\right),$$

respectively, where $x\left(t\right)={\left[x_{-2N}\left(t\right),x_{-2N+1}\left(t\right),\cdots ,x_{0}\left(t\right),\cdots ,x_{2N}\left(t\right)\right]}^{T}$ is the M×1 subarray output of x-axis, $a_{x}={\left[e^{j\tau_{x,-2N}},\cdots ,1,\cdots ,e^{j\tau_{x,2N}}\right]}^{T}$ denotes the subarray manifold of x-axis, and $n_{x}\left(t\right)={\left[n_{x,-2N}\left(t\right),\cdots ,n_{0},\cdots ,\;\mathrm{and}\;n_{x,2N}\left(t\right)\right]}^{T}$ is the noise part on the subarray of *x*-axis. Likewise, $y\left(t\right)={\left[y_{-2N}\left(t\right),y_{-2N+1}\left(t\right),\cdots ,y_{0}\left(t\right),\cdots ,y_{2N}\left(t\right)\right]}^{T}$, $a_{y}={\left[e^{j\tau_{y,-2N}},\cdots ,1,\cdots ,e^{j\tau_{y,2N}}\right]}^{T}$ and $n_{y}\left(t\right)={\left[n_{y,-2N}\left(t\right),\cdots ,n_{y,2N}\left(t\right)\right]}^{T}$ are defined as the subarray output, subarray direction matrix, and noise matrix on the *y*-axis, respectively.

Under the above conditions, by combining $x\left(t\right)$ and $y\left(t\right)$, the array output is given by

(11) $$z\left(t\right)=\left[\begin{array}{c} x\left(t\right)\\ y\left(t\right) \end{array}\right]=as\left(t\right)+n\left(t\right)$$

where $a={\left[a_{x}^{T},a_{y}^{T}\right]}^{T}\in C^{2M\times 1}$ represents the direction matrix of the array and $n\left(t\right)=[n_{x}^{T}\left(t\right),$ $n_{y}^{T}\left(t\right){]}^{T}$.

## 3. RD-MUSIC Algorithm

The 3D-MUSIC algorithm requires a joint parameter estimation of the elevation, azimuth, and range, which is extremely time consuming, thus it is necessary to reduce its complexity by the reduced-dimension method. Based on this, a two-stage reduced-dimension approach is provided in the RD-MUSIC algorithm, and the steps of the algorithm are described in detail below.

### 3.1. Relationships of the Direction Matrices

In order to obtain the relationships between the direction matrices $a_{x}$ and $a_{y}$ of the two subarrays, the eigenvalue decomposition of the covariance matrix R for the array output matrix is first performed as

(12) $$R=E[z\left(t\right)z^{H}\left(t\right)]=u_{s}\Gamma_{s}u_{s}^{H}+u_{n}\Gamma_{n}u_{n}^{H}$$

where $\Gamma_{s}$ is a diagonal matrix whose diagonal elements contain only one large eigenvalue in R, and the diagonal matrix $\Gamma_{n}$ contains the remaining small eigenvalues in R; $u_{s}$ and $u_{n}$ are regarded as the signal subspace and noise subspace, which are matrices composed of eigenvectors corresponding to the eigenvalues in $\Gamma_{s}$ and $\Gamma_{n}$, respectively.

The signal subspace and the array direction matrix span in the same subspace [23], so we can obtain $u_{s}={\left[\begin{array}{cc} u_{sx}^{T} & u_{sy}^{T} \end{array}\right]}^{T}={\left[\begin{array}{cc} a_{x}^{T} & a_{y}^{T} \end{array}\right]}^{T}\varepsilon$, where ε is a non-zero complex number. Then, two connection matrices can be constructed as $H_{x}=u_{sy}u_{sx}^{+}$ and $H_{y}=u_{sx}u_{sy}^{+}$. By simple substitution, we can have $H_{x}=a_{y}\varepsilon \varepsilon^{-1}a_{x}^{+}$ and $H_{y}=a_{x}\varepsilon \varepsilon^{-1}a_{y}^{+}$. Finally, the relationships of the direction matrices on the x-axis and the y-axis can be obtained as

(13) $$a_{y}=H_{x}a_{x}$$

(14) $$a_{x}=H_{y}a_{y}$$

From the above equations, it can be seen that the relationships of the two direction matrices are extremely simple, which will help to reduce the dimensionality of the 3D-MUSIC algorithm presented in the following subsection.

### 3.2. Classical MUSIC Algorithm

According to the subspace theory of array signal processing, the signal and the noise subspaces are orthogonal to each other. The classical 3D-MUSIC spectral function of the single source can be represented by

(15) $$f_{3D-\mathrm{MUSIC}}\left(\theta ,\varphi ,r\right)=\frac{1}{a^{H}\left(\theta ,\varphi ,r\right)U_{n}U_{n}^{H}a\left(\theta ,\varphi ,r\right)}$$

where $a\left(\theta ,\varphi ,r\right)={\left[a_{x}^{T},a_{y}^{T}\right]}^{T}$. In order to obtain the paired angles and range information of the source, a joint spectral search over the angle and range domains is performed to yield the maximum result of $f_{3D-\mathrm{MUSIC}}$, which is expressed as

(16) $$\left(\hat{\theta },\hat{\varphi },\hat{r}\right)=\underset{\theta ,\varphi ,r}{\arg\max}f_{3D-\mathrm{MUSIC}}\left(\theta ,\varphi ,r\right)$$

To solve the optimization of the above problem, the estimation of θ, φ, and *r* can be obtained by traversing the values of angles and range in possible space and searching the peak of the spectral function. However, a multidimensional searching for multiple parameters is required in practice, which is tremendously time-consuming.

### 3.3. Reduced Dimensional Search

To reduce the computational complexity of the multidimensional search, the first dimension reduction can be performed with the help of two connection matrices, $H_{x}$ and $H_{y}$ in (13) and (14). Through simple derivation, the spectral function $f_{3D-\mathrm{MUSIC}}$ can be reconstructed as

(17) $$f_{1}\left(\gamma_{x},\phi_{x}\right)=\frac{1}{a_{x}^{H}\left(\gamma_{x},\phi_{x}\right)G_{x}G_{x}^{H}a_{x}\left(\gamma_{x},\phi_{x}\right)}$$

(18) $$f_{2}\left(\gamma_{y},\phi_{y}\right)=\frac{1}{a_{y}^{H}\left(\gamma_{y},\phi_{y}\right)G_{y}G_{y}^{H}a_{y}\left(\gamma_{y},\phi_{y}\right)}$$

where $G_{x}=\left[\begin{array}{cc} I_{M} & H_{x}^{H} \end{array}\right]U_{n}$ and $G_{y}=\left[\begin{array}{cc} H_{y}^{H} & I_{M} \end{array}\right]U_{n}$. Hence, the 3-D parameter estimation is converted to two 2-D estimations, which decreases the computation.

Due to the symmetry of the array structure, the direction matrix of the subarray on the x-axis is decomposed into the multiplication of two matrices, $\Gamma \left(\gamma_{x}\right)\in C^{M\times \left(2N+1\right)}$ and $k\left(\phi_{x}\right)\in C^{\left(2N+1\right)\times 1}$, as shown in (19) on the top of next page, where $\Gamma \left(\gamma_{x}\right)$ contains DOA information of the source whereas $k\left(\phi_{x}\right)$ includes both DOA and range information. Similarly, the direction matrix of the subarray on the y-axis is given in (20). It is observed from (19) and (20) that the angle components are completely independent of each other.

(19) $$a_{x}\left(\gamma_{x},\phi_{x}\right)=\begin{array}{c} \underset{\underset{\Gamma \left(\gamma_{x}\right)}{︸}}{\left[\begin{array}{cccc} e^{j\gamma_{x}l_{x,-2N}} & & & \\ 0 & e^{j\gamma_{x}l_{x,-2N+1}} & & \\ & 0 & \ddots & \\ \vdots & \vdots & 0 & 1\\ & 0 & ⋰ & \\ 0 & e^{j\gamma_{x}l_{x,-2N+1}} & & \\ e^{j\gamma_{x}l_{x,-2N+1}} & & & \end{array}\;\right]} \end{array}\times \begin{array}{c} \underset{\underset{k\left(\phi_{x}\right)}{︸}}{\left[\begin{array}{c} e^{j\phi_{x}l_{x,2N}^{2}}\\ e^{j\phi_{x}l_{x,2N-1}^{2}}\\ \vdots \\ 1 \end{array}\right]} \end{array}$$

(20) $$a_{y}\left(\gamma_{x},\phi_{x}\right)=\begin{array}{c} \underset{\underset{\Gamma \left(\gamma_{y}\right)}{︸}}{\left[\begin{array}{cccc} e^{j\gamma_{y}l_{y,-2N}} & & & \\ 0 & e^{j\gamma_{y}l_{y,-2N}} & & \\ & 0 & \ddots & \\ \vdots & \vdots & 0 & 1\\ & 0 & ⋰ & \\ 0 & e^{j\gamma_{y}l_{y,-2N}} & & \\ e^{j\gamma_{y}l_{y,-2N}} & & & \end{array}\;\right]} \end{array}\times \begin{array}{c} \underset{\underset{k\left(\phi_{y}\right)}{︸}}{\left[\begin{array}{c} e^{j\phi_{y}l_{y,2N}^{2}}\\ e^{j\phi_{y}l_{y,2N-1}^{2}}\\ \vdots \\ 1 \end{array}\right]} \end{array}$$

Through substituting (19) into (17), the spectral function in (17) can be rewritten as

(21) $$f_{1}\left(\gamma_{x},\phi_{x}\right)=\frac{1}{k^{H}\left(\phi_{x}\right)Q_{x}\left(\gamma_{x}\right)k\left(\phi_{x}\right)}$$

where $Q_{x}=\Gamma^{H}\left(\gamma_{x}\right)G_{x}G_{x}^{H}\Gamma \left(\gamma_{x}\right)$. Similarly, the spectral peak search function $f_{2}$ can be expressed as

(22) $$f_{2}\left(\gamma_{y},\phi_{y}\right)=\frac{1}{k^{H}\left(\phi_{y}\right)Q_{y}\left(\gamma_{y}\right)k\left(\phi_{y}\right)}$$

where $Q_{y}=\Gamma^{H}\left(\gamma_{y}\right)G_{y}G_{y}^{H}\Gamma \left(\gamma_{y}\right)$.

According to (21) and (22), it can be concluded that the highest peaks of $f_{1}$ and $f_{2}$ correspond to the estimated values of the source elevation angle, azimuth angle, and range.

The spectral peak search function $f_{1}\left(\gamma_{x},\phi_{x}\right)$ has the maximum value, which is equivalent to solving the following quadratic optimization problem

(23) $$\min_{\gamma_{x}}\;\;\;\;\;V\left(\gamma_{x},\phi_{x}\right)\;,\;\;\;\;\;\;\;\;\;\;\;\;\;\;\;\;\;\;\;\;\;\;\;\mathrm{s.t}\;\;\;\;\;\;p^{H}k\left(\phi_{x}\right)\;\;=1$$

where $V\left(\gamma_{x},\phi_{x}\right)=k^{H}\left(\phi_{x}\right)Q_{x}\left(\gamma_{x}\right)k\left(\phi_{x}\right)$ and $p={\left[0,0,\cdots ,0,1\right]}^{T}\in R^{(2N+1)\times 1}$. The condition $p^{H}k\left(\phi_{x}\right)\;\;=1$ is employed to eliminate the trivial solution of the equation $k\left(\phi_{x}\right)=O_{2N+1}$.

Furthermore, it is suitable for Lagrange’s multiplier method to determine the minimum problems subject to a equality constraint, which will convert the constrained problem to an unconstrained one. The Lagrangian function of (23) is given by

(24) $$L\left(\gamma_{x},\phi_{x}\right)=k^{H}\left(\phi_{x}\right)Q_{x}\left(\gamma_{x}\right)k\left(\phi_{x}\right)-\omega (p^{H}k\left(\phi_{x}\right)-1)$$

where ω is the Lagrange multiplier. Taking the partial derivative of $k\left(\phi_{x}\right)$ in (24), we can obtain

(25) $$\frac{\partial L\left(\gamma_{x},\phi_{x}\right)}{\partial k\left(\phi_{x}\right)}=2Q_{x}\left(\gamma_{x}\right)k\left(\phi_{x}\right)+\omega p=0$$

By solving (25) and combining the constrained condition $p^{H}k\left(\phi_{x}\right)=1$, the vector $k\left(\phi_{x}\right)$ can be represented by $Q_{x}\left(\gamma_{x}\right)$, which is given by

(26) $$k\left(\phi_{x}\right)=\frac{Q_{x}{\left(\gamma_{x}\right)}^{-1}p}{p^{H}Q_{x}{\left(\gamma_{x}\right)}^{-1}p}$$

This relationship in (26) will help to simplify the quadratic optimization problem in (23). By inserting $k\left(\phi_{x}\right)$ of (26) into (23), the quadratic optimization problem in (23) can be rewritten as

(27) $${\hat{\gamma }}_{x}=\;\underset{\gamma_{x}}{\arg\max}\;p^{H}Q_{x}{\left(\gamma_{x}\right)}^{-1}p$$

where $\gamma_{x}\in \left(-\pi /2,\pi /2\right)$. To solve the optimum value of $\gamma_{x}$, exhaustive searching is a common method in the MUSIC algorithm, which traverses all the values of $\gamma_{x}$ in possible values and searches the peak of the optimization function in (27). Moreover, this searching process is one 1-D search, which requires low computation.

In a similar way, $\hat{\gamma_{y}}$ can also be obtained by exhaustive searching, as

(28) $${\hat{\gamma }}_{y}=\;\underset{\gamma_{y}}{\arg\max}\;p^{H}Q_{y}{\left(\gamma_{y}\right)}^{-1}p$$

where $\gamma_{y}\in \left(-\pi /2,\pi /2\right)$.

Therefore, the two 2-D search in (21) and (22) are transformed into two 1-D estimations for $\gamma_{x}$ and $\gamma_{y}$ in (27) and (28), respectively. By simple mathematical operations, the elevation angle and azimuth angle of the source can be given, respectively, by

(29) $$\hat{\varphi }=\arctan\left(\frac{{\hat{\gamma }}_{y}}{{\hat{\gamma }}_{{}_{x}}}\right)$$

(30) $$\hat{\theta }=\arcsin\left(\frac{\lambda }{2\pi d}\sqrt{{{\hat{\gamma }}_{x}}^{2}+{{\hat{\gamma }}_{y}}^{2}}\right)$$

To this end, the two-stage dimension reduction is completed, and the angle estimates of the single source can be acquired by performing two 1-D searches.

By substituting the estimated elevation and azimuth of (29) and (30) into (16), the spectral function of the range of the source can be obtained as

(31) $$\begin{array}{cc} \hat{r}= & \underset{r}{\mathrm{argmax}}f_{3D-\mathrm{MUSIC}}\left(\hat{\theta },\hat{\varphi },r\right)\\ = & \underset{r}{\mathrm{argmax}}\frac{1}{a^{H}\left(\hat{\theta },\hat{\varphi },r\right)U_{n}U_{n}^{H}a\left(\hat{\theta },\hat{\varphi },r\right)} \end{array}$$

where r∈[0.62(D3D3λλ)1/2,2D22D2λλ], and *D* denotes the array aperture.

Through exhaustive searching over the possible range space, the estimate of the source range can be obtained by finding the peak of the spectral function over all possible values of *r*, which utilizes the orthogonality of the noise eigenvector and the signal vector.

In summary, it can be found that the proposed RD-MUSIC algorithm only requires three 1-D searches and fully utilizes both the signal and noise subspaces, whereas the ESPRIT and MUSIC algorithms utilize either the signal or noise subspace. Moreover, the main steps of the proposed source localization algorithm are summarized as follows.

- Step 1. Calculate the covariance matrix of the array, $\hat{R}=\frac{1}{J}\sum_{t=1}^{J}z\left(t\right)z^{H}\left(t\right)$, where *J* denotes the number of snapshots, and perform eigenvalue decomposition operation on $\hat{R}$ to obtain the signal subspace $U_{s}$ and noise subspace $U_{n}$.
- Step 2. Use (13) and (14) to obtain the relationships in the two direction matrices of the two subarrays.
- Step 3. Perform two 1-D spectral search of (27) and (28) to obtain ${\hat{\gamma }}_{x}$ and ${\hat{\gamma }}_{y}$, respectively, and calculate the elevation angle and azimuth angle through (29) and (30).
- Step 4. Based on $\hat{\varphi }$ and $\hat{\theta }$, the range result can be obtained by conducting a 1-D spectral search on (31).

## 4. Complexity Analysis

For 3-D source localization, the computational complexity is one of the important evaluation criterion. The evaluation unit of computational complexity is the number of multiplications involved in the algorithm. With the SSCA structure, the computational complexity comparisons, among the proposed RD-MUSIC, the RARE-MUSIC [16], and the 3D-MUSIC, are given in Table 2.

The complexity of the RD-MUSIC algorithm mainly depends on two major processes: angle estimations and range estimation, denoted as $2n_{e}\left[\left(\left(3M-1\right)M^{2}+\left(M+1\right)\right)/2+3{\left(M+1\right)}^{3}/8\right]+{\left(2M\right)}^{2}J+{\left(2M\right)}^{3}$ and $n_{l}\left(2M\right)\left[{\left(2M\right)}^{2}+1\right]$, respectively, where ne=ππΔΔ represents the number of spectral peak searches in the angle domain, $n_{l}$ represents the number of spectral peak searches in the distance interval, and Δ is the search step.

**Figure 2:**
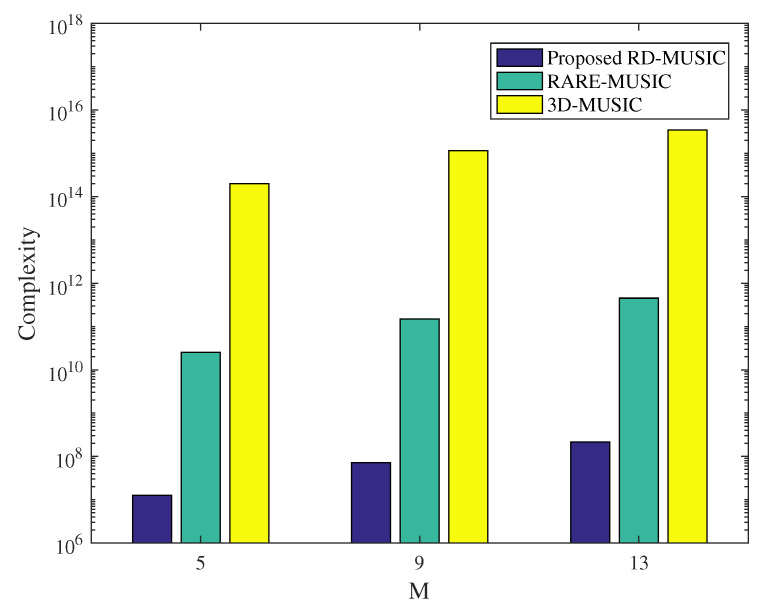
Comparison of the complexity of algorithms.

Figure 2 exhibits the complexity comparison of three different algorithms with the SSCA, where J=200, a=3, b=4, and Δ=0.001. It can be obviously found that the complexity of the traditional 3D-MUSIC algorithm is much higher than that of the other two algorithms due to $n_{e}^{2}n_{l}\gg n_{e}^{2}+n_{l}$, under the same array structure. Moreover, the complexity of the first part of the proposed RD-MUSIC is mainly caused by the two 1-D search for angles, which is significantly lower than that of the RARE-MUSIC. Moreover, although both the RD-MUSIC and RARE-MUSIC algorithms separate the angle and range terms in the same manner, the global complexity of the former is lower than that of the latter. This is because only two 1-D searches are required for the elevation and azimuth angles in the proposed RD-MUSIC algorithm, whereas a 2-D joint search for these two angles is needed in the RARE-MUSIC algorithm.

## 5. Simulation Results

In this section, comprehensive simulations to verify the validity of the proposed RD-MUSIC algorithm are provided. The root mean square errors (RMSEs) of the estimation for angles and range are used as the evaluating indicators of the performance, which are given, respectively, by

(32) $${\mathrm{RMSE}}_{\mathrm{angle}}\;=\sqrt{\frac{1}{U}\sum_{u=1}^{U}\left[{\left(\hat{\theta }-\theta \right)}^{2}+{\left(\hat{\varphi }-\varphi \right)}^{2}\right]}$$

(33) $${\mathrm{RMSE}}_{\mathrm{angle}}\;=\sqrt{\frac{1}{U}\sum_{u=1}^{U}{\left(\hat{r}-r\right)}^{2}}$$

where *U* denotes the number of Monte-Carlo trials; $\hat{\theta }$, $\hat{\varphi }$, and $\hat{r}$ represent the estimated values of the *u*-th Monte-Carlo trial of θ, φ, and *r*, respectively. For each simulation scenario, U=1000 rounds of Monte-Carlo runs are conducted.

To further elaborate on the performance of the proposed algorithm, the transmitted signal with a carrier frequency of $f_{c}=2.45$ GHz is used, and the corresponding wavelength of the narrowband signal is λ=0.1224 m. The parameters for the sparse cross array are with M=5, a=3, and b=4. Therefore, the subarray aperture is D=8d. Here, a single source is located at $\left(\theta ,\varphi ,r\right)=\left(35.3^{\circ },58.6^{\circ },3\lambda \right)$, and the searching step is Δ=0.001.

**Figure 3:**
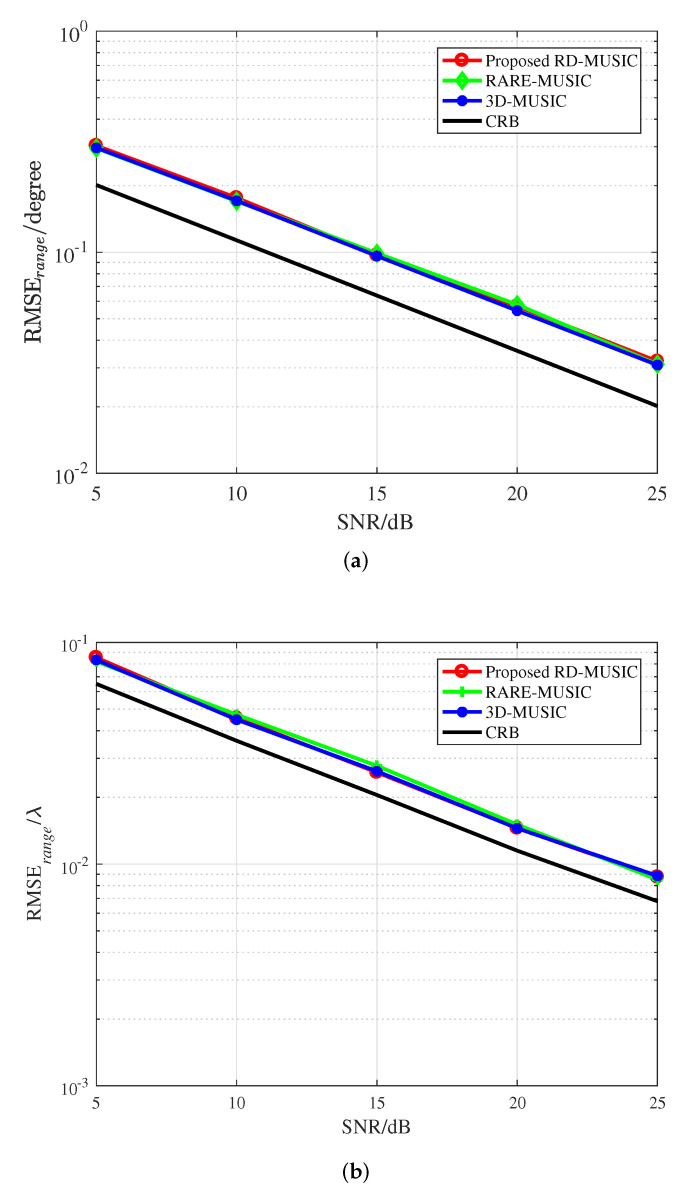
RMSEs versus SNR. (**a**) DOA estimation. (**b**) Range estimation.

Figure 3 illustrates the performance of the proposed algorithm in comparison with RARE-MUSIC, 3D-MUSIC, and the Cramer-Rao Bound, under the condition of the same array structure, where J=200. It can be seen that the curves of the three algorithms almost overlap with each other, which means that the proposed algorithm with much lower complexity can achieve the similar performance with the 3D-MUSIC and RARE-MUSIC algorithms and approach the CRB as the SNR increases.

**Figure 4:**
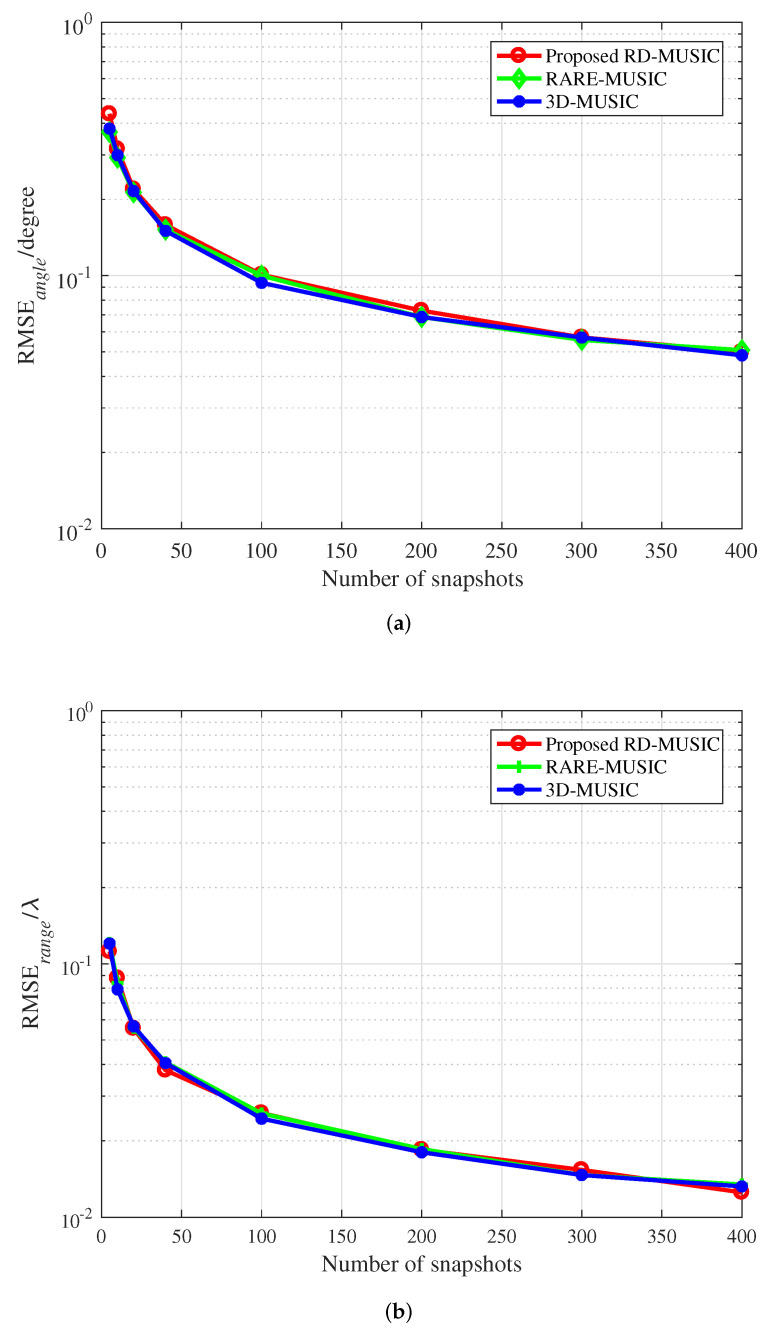
RMSEs versus the number of snapshots. (**a**) DOA estimation. (**b**) Range estimation.

Figure 4 indicates the estimation performance of algorithms under different snapshots when SNR = 18 dB. It can be found that the estimation performance of the proposed algorithm will be improved as the number of snapshots increases. From Figure 3 and Figure 4, the statistical performance of the proposed algorithm based on the MUSIC can almost reach that of 3D-MUSIC estimation.

**Figure 5:**
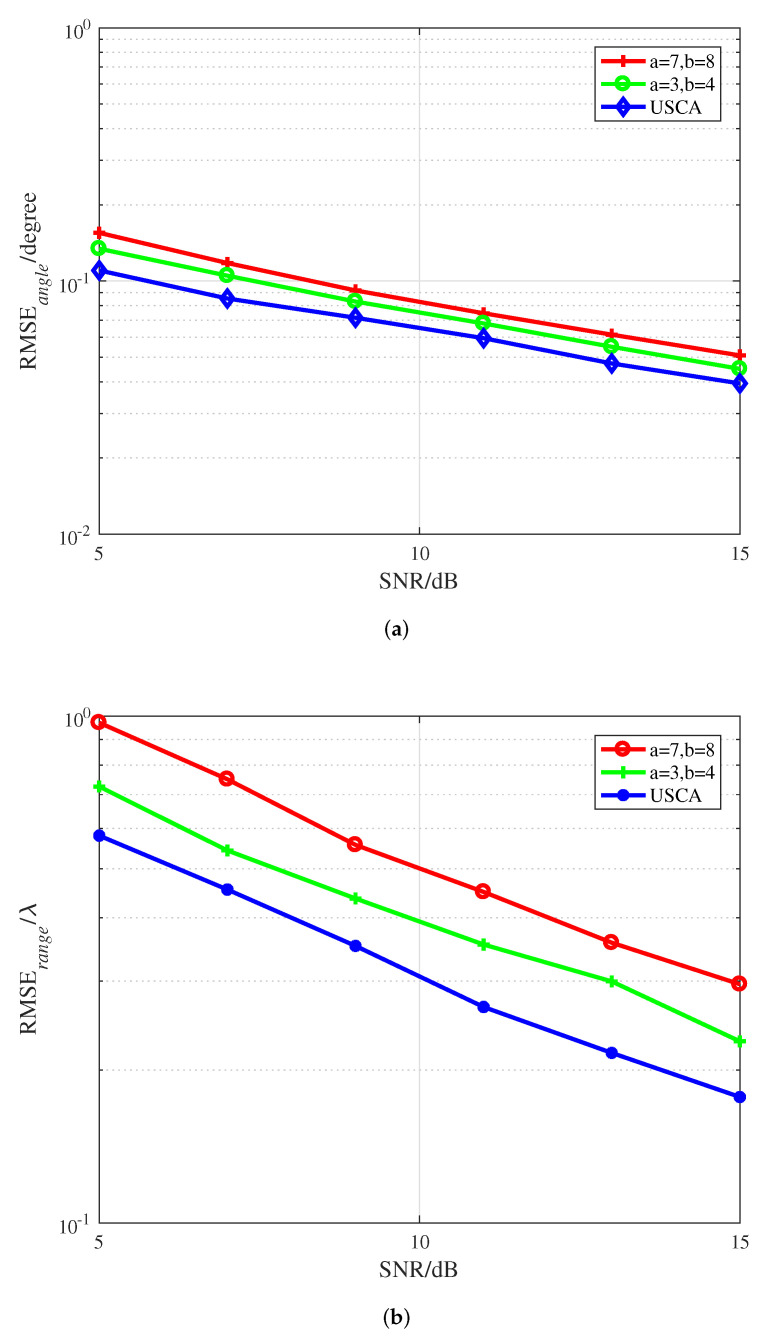
RMSEs versus SNR under different coprime factor pairs with the same subarray aperture. (**a**) DOA estimation. (**b**) Range estimation.

In order to evaluate the robustness of the proposed algorithm, its performance with uniform inter-element spacing *d* and coprime inter-element spacing is compared for the identical subarray aperture, as shown in Figure 5, where the aperture of the subarray for each axis is D=16d, the coprime factor pairs are $a_{1}=7$, $b_{1}=8$, and $a_{2}=3$, $b_{2}=4$, and the source is located at $\left(\theta ,\varphi ,r\right)=\left(35.3^{\circ },58.6^{\circ },20\lambda \right)$. Estimation results for the SSCA structure and USCA structure are presented in Figure 5, where it can be seen that the proposed SSCA outperforms the USCA with the same subarray aperture. Moreover, when the array aperture is fixed, the larger the coprime factor pair is, the smaller the number of array elements is, and also the lower the measurement accuracy is.

Because the results of (32) cannot analyze the independent effects of the elevation and azimuth on the DOA estimation results for the proposed algorithm, the RMSEs of the elevation and azimuth angles are separated to further illustrate the DOA estimation at any location in space, as

(34) $${\mathrm{RMSE}}_{\alpha }=\sqrt{\frac{1}{U}\sum_{u=1}^{U}{\left(\hat{\alpha }-\alpha \right)}^{2}}$$

where α represents the real value θ or φ, and $\hat{\alpha }$ denotes the estimated value $\hat{\theta }$ or $\hat{\varphi }$. In the simulations, SNR = 18 dB and J=200.

**Figure 6:**
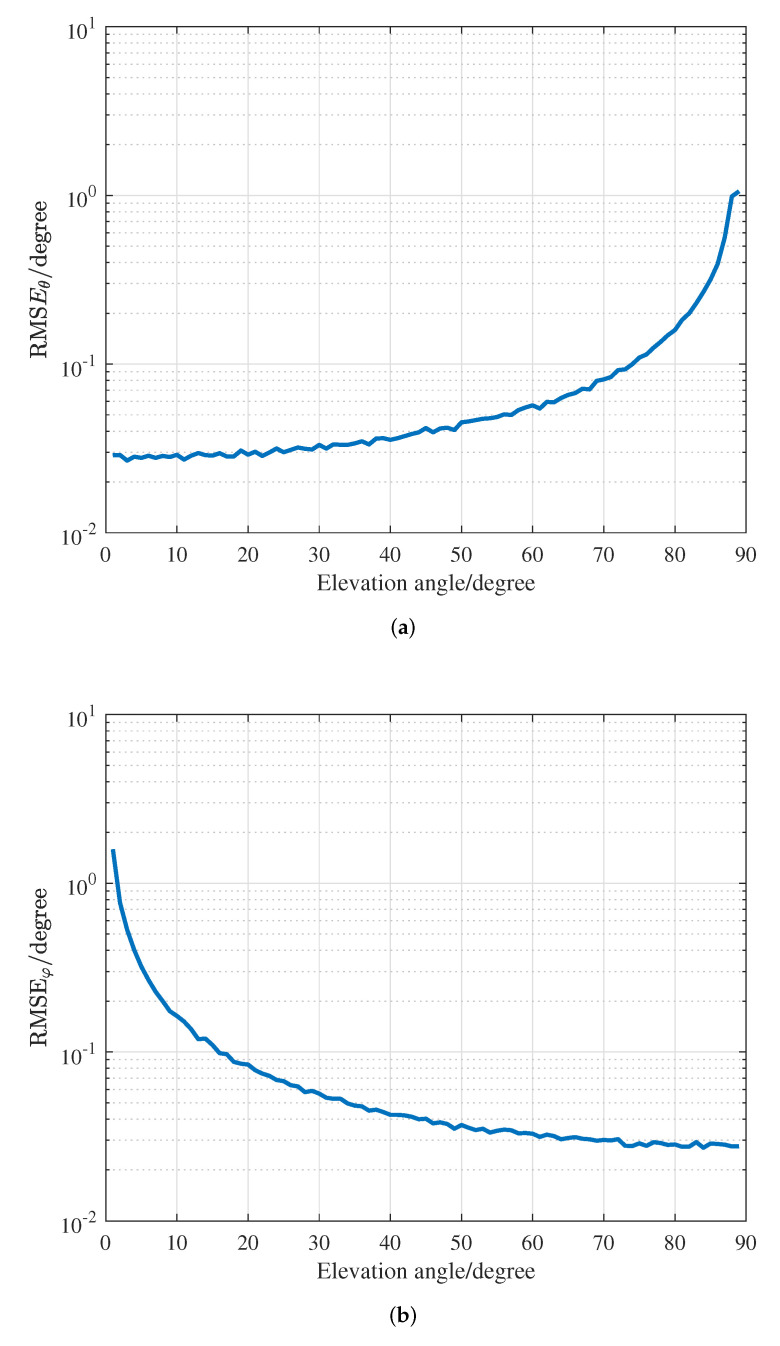
RMSEs versus the elevation angle when $\varphi =45^{\circ }$. (**a**) RMSE of elevation. (**b**) RMSE of azimuth.

Figure 6 indicates the effect of the elevation angle variation on the performance of the proposed algorithm, where the azimuth angle is $45^{\circ }$ and the elevation angle of the source varies from $1^{\circ }$ to $89^{\circ }$. It can be clearly found from Figure 6a that the estimation performance of the elevation angle becomes worse as the elevation angle of the source increases, since the resolution of the SSCA structure decreases when the direction of the source is close to the plane of the antenna array. In Figure 6b, on the contrary, the estimation performance of the azimuth angle of the source is opposite to that of the elevation angle. In other words, as the elevation angle of the source increases, the estimation performance of the azimuth angle is improved. This is because the equivalent aperture of the CA structure is augmented in the azimuthal direction when the direction of the source is close to the plane of the antenna array.

**Figure 7:**
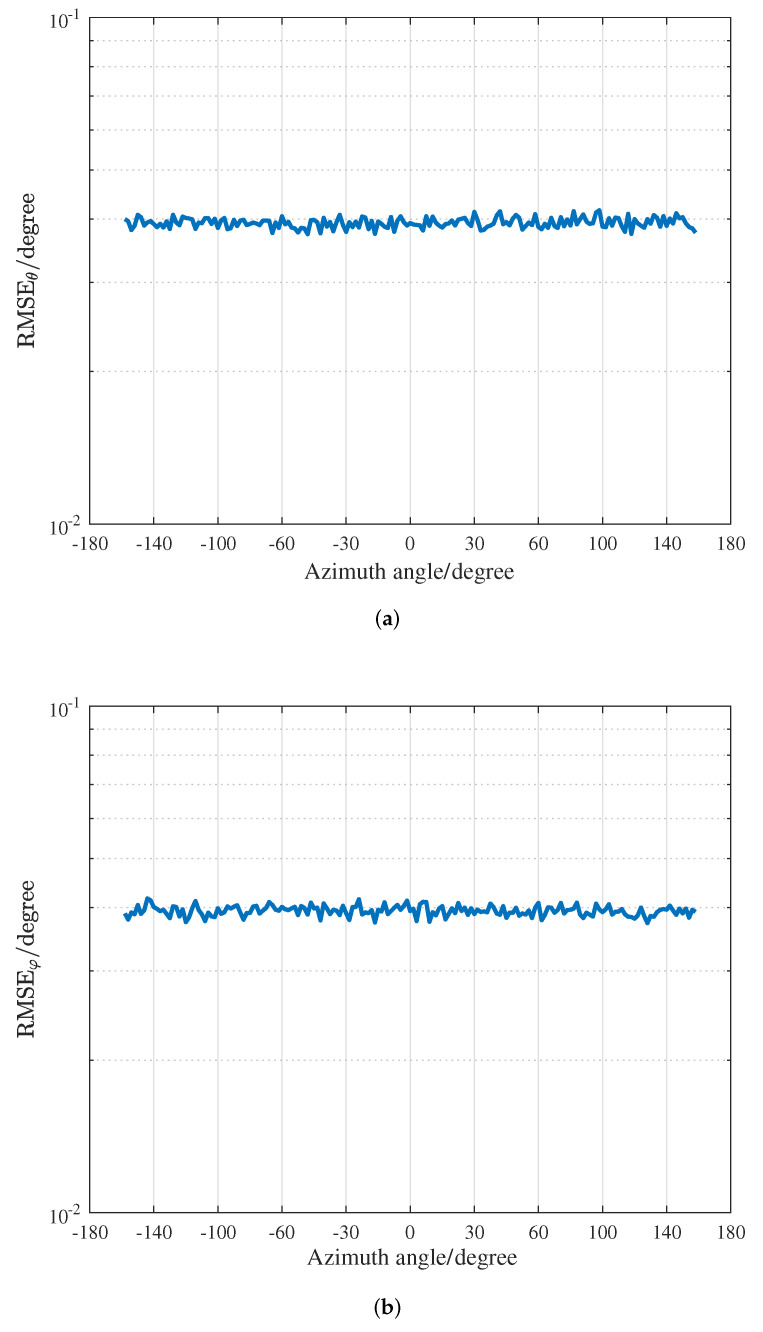
RMSEs versus the azimuth angle when $\theta =45^{\circ }$. (**a**) RMSE of elevation. (**b**) RMSE of azimuth.

Figure 7 presents the effect of the azimuth angle variation on the performance of proposed algorithm, where the elevation of the source is $45^{\circ }$ and the azimuth angle of the source varies from $-179^{\circ }$ to $179^{\circ }$. It can be found that the SSCA structure has almost the identical estimation accuracy for the elevation and azimuth angles when the azimuth angle changes. The effect of the cross array geometry on the DOA estimation results is analyzed, which facilitates the design and deployment of practical applications.

## 6. Conclusions

In this article, a low-complexity 3-D localization algorithm, RD-MUSIC, was presented for the single source, where the SSCA structure is designed to further reduce complexity. By exploiting the two-stage reduced-dimension method, the classical 3-D parameter estimation of MUSIC is converted into three 1-D estimations, where two 1-D searches are applied to estimate the angles, and the other 1-D MUSIC is utilized to estimate the range of the source. Compared with the classical 3D-MUSIC and RARE-MUSIC algorithms, the computational complexity of the proposed RD-MUSIC is greatly reduced, especially when employing the SSCA structure. Simulation results show that the performance of the proposed low-complexity algorithm is very similar to the performances of the RARE-MUSIC and 3D-MUSIC algorithms, which makes the RD-MUSIC algorithm a better choice in practical applications.

## Figures and Tables

**Table 1:** Notations in this article.

Notation	Definition
${\left(.\right)}^{T}$	Transposition operation
${\left(.\right)}^{H}$	Conjugate transpose operation
${\left(.\right)}^{+}$	Pseudo-inversion operation
${\left(.\right)}^{-1}$	Inversion operation
E	Statistical expectation
$I_{M}$	An *M*-by-*M* identity matrix
$O_{2N+1}$	An $\left(2N+1\right)$-by-$\left(2N+1\right)$ null matrix

**Table 2:** Computational complexity of algorithms.

Algorithm	Complexity
Proposed RD-MUSIC	$\begin{array}{c} \;\;\;{\left(2M\right)}^{2}J\;+\;\;{\left(2M\right)}^{3}\;+\;\;n_{l}\left(2M\right)\left[{\left(2M\right)}^{2}+1\right]\;+\;\;2n_{e}\left[(\left(3M\;-\;\;1\right)M^{2}\;+\;\;\left(M+1\right))/2\;+\;\;3{\left(M\;+\;\;1\right)}^{3}/8\right] \end{array}$
RARE-MUSIC	$\begin{array}{c} \;\;\;{\left(2M\right)}^{2}J+{\left(2M\right)}^{3}+n_{l}\left(2M\right)\left[{\left(2M\right)}^{2}+1\right]+2n_{e}^{2}\left[{\left(2M\right)}^{2}\left(5M-1\right)/2+M{\left(M+1\right)}^{2}/2\right] \end{array}$
3D-MUSIC	${\left(2M\right)}^{2}J+{\left(2M\right)}^{3}+2n_{e}^{2}n_{l}\left(2M\right)\left[{\left(2M\right)}^{2}+1\right]$

## Data Availability

Not applicable.
